# Pathways from Trauma to Psychotic Experiences: A Theoretically Informed Model of Posttraumatic Stress in Psychosis

**DOI:** 10.3389/fpsyg.2017.00697

**Published:** 2017-05-23

**Authors:** Amy Hardy

**Affiliations:** ^1^Institute of Psychiatry, Psychology & Neuroscience, King’s College LondonLondon, UK; ^2^Psychosis Clinical Academic Group, South London and Maudsley NHS Foundation TrustLondon, UK

**Keywords:** trauma, psychosis, posttraumatic stress, emotion regulation, perceptual memory, episodic memory, personal semantic memory, intrusions

## Abstract

In recent years, empirical data and theoretical accounts relating to the relationship between childhood victimization and psychotic experiences have accumulated. Much of this work has focused on co-occurring Posttraumatic Stress Disorder or putative causal mechanisms in isolation from each other. The complexity of posttraumatic stress reactions experienced in psychosis remains poorly understood. This paper therefore attempts to synthesize the current evidence base into a theoretically informed, multifactorial model of posttraumatic stress in psychosis. Three trauma-related vulnerability factors are proposed to give rise to intrusions and to affect how people appraise and cope with them. First, understandable attempts to survive trauma become habitual ways of regulating emotion, manifesting in cognitive-affective, behavioral and interpersonal responses. Second, event memories, consisting of perceptual and episodic representations, are impacted by emotion experienced during trauma. Third, personal semantic memory, specifically appraisals of the self and others, are shaped by event memories. It is proposed these vulnerability factors have the potential to lead to two types of intrusions. The first type is anomalous experiences arising from emotion regulation and/or the generation of novel images derived from trauma memory. The second type is trauma memory intrusions reflecting, to varying degrees, the retrieval of perceptual, episodic and personal semantic representations. It is speculated trauma memory intrusions may be experienced on a continuum from contextualized to fragmented, depending on memory encoding and retrieval. Personal semantic memory will then impact on how intrusions are appraised, with habitual emotion regulation strategies influencing people’s coping responses to these. Three vignettes are outlined to illustrate how the model accounts for different pathways between victimization and psychosis, and implications for therapy are considered. The model is the first to propose how emotion regulation and autobiographical memory may lead to a range of intrusive experiences in psychosis, and therefore attempts to explain the different phenomenological associations observed between trauma and intrusions. However, it includes a number of novel hypotheses that require empirical testing, which may lead to further refinement. It is anticipated the model will assist research and practice, in the hope of supporting people to manage the impact of victimization on their lives.

## Introduction

It seems obvious that what happens in our lives has the potential to shape how we are. And yet, for so long in mental healthcare, the life stories of people with psychosis have been neglected. A wealth of empirical evidence now questions this position, highlighting the significant impact that traumatic events^1^, particularly childhood victimization, can have on people’s difficulties (Morgan and Gayer-Anderson, 2016). The challenge, therefore, is to reduce the impact of adversity on the lives of people with psychosis. Necessary steps include a focus on prevention, and a humane approach to identification, safeguarding and redress (Read and Bentall, 2012). Alongside this, effective interventions are needed to support people to manage the consequences of victimization and move on in life. The development of trauma-focused therapy for psychosis is dependent on an evidence-based understanding of the pathways from trauma to psychotic experiences. This understanding can then be used to highlight targets and strategies for intervention, in line with the interventionist causal model (Kendler and Campbell, 2009). But this is no easy task. The diverse phenomenology of psychosis indicates these pathways are complex; they will vary from person to person and fluctuate over time. Nonetheless, progress is being made in identifying possible underlying mechanisms and theoretical models have been proposed.

This paper outlines a multifactorial model that aims to integrate existing accounts, drawing on cognitive-behavioral, attachment and neuropsychological perspectives of psychosis and PTSD. Within the model, these accounts are viewed as complimentary, in that both cognitive-behavioral and attachment approaches highlight the importance of internal representations of experience, the meaning derived from these experiences, the ways emotions are regulated, and how these mechanisms interact with neuropsychological processes. A central role is therefore proposed for autobiographical memory and trauma-related emotion regulation strategies in shaping the phenomenology of intrusive imagery, subsequent appraisals and coping responses. Evidence and accounts of the relationships between trauma and psychosis will first be briefly reviewed, then the new model of posttraumatic stress in psychosis described. Three vignettes, based on clinical experience, will be discussed in relation to the model to illustrate the possible ways psychological posttraumatic stress processes may play a role in psychosis. Implications for intervention indicated by the model will be reflected on, together with methodological challenges and directions for future research.

## Relationships Between Trauma and Psychosis

The relationship between trauma and psychosis is now well established. Higher rates of childhood victimization and Posttraumatic Stress Disorder (PTSD, DSM 5, American Psychiatric Association, 2013) are reported across the spectrum of psychosis compared to the general population, although findings vary depending on the population sampled and methodology used (Kessler et al., 2005; Achim et al., 2011; Matheson et al., 2013; Kraan et al., 2015; Steel et al., 2017a). The most robust study of PTSD prevalence estimated 16% of people with schizophrenia-spectrum diagnoses meet diagnostic criteria (de Bont et al., 2015). This is of relevance to mental healthcare as co-occurring PTSD and psychosis appear to be associated with worse clinical and functional outcomes, and difficulties with engagement, adherence, and treatment response (Schneeberger et al., 2014; Hassan and De Luca, 2015; Mondelli et al., 2015; Trotta et al., 2015; Seow et al., 2016).

Given the higher incidence of trauma in psychosis, research has considered whether victimization may play a causal role in psychotic experiences, at least for some people. Demonstrating the causality of trauma in psychosis is methodologically complex and research is in its infancy. However, preliminary findings are in line with the proposal that victimization does have a causal effect. Prospective, case-control, and epidemiological studies support an association, and dose-response relationships and mediation by plausible mechanisms have been demonstrated (Hill, 1965; Varese et al., 2012b; Kelleher et al., 2013; Alsawy et al., 2015; Hardy et al., 2016; Amanuel et al., 2017; McGrath et al., 2017).

A key controversy is whether victimization has a generic impact on mental health outcomes or if there is some degree of specificity between certain trauma types and symptoms of psychosis (Bentall et al., 2014; van Nierop et al., 2014a, 2015; van Dam et al., 2015). The latter hypothesis is based on the possibility that some events may have a greater propensity to trigger particular psychological mechanisms. For example, it has been found that childhood sexual abuse is associated with voices, whereas emotional abuse and neglect are related to paranoia, with the former at least partially accounted for by emotion regulation and the latter by beliefs about the self and others (Sitko et al., 2014; Hardy et al., 2016; Wickham and Bentall, 2016). However, a consensus on the debate has yet to be reached and further investigation is needed.

From a symptom-specific perspective, the association between victimization and positive symptoms has been more established than for negative symptoms (Bentall et al., 2014). Relationships between trauma and negative symptoms are feasible, given the proposal they represent a response to the psychologically overwhelming trauma of psychosis, together with the phenomenological overlap between them and the avoidance and numbing symptoms of PTSD (Stampfer, 1990). However, current findings are equivocal (Resnick et al., 2003; Lysaker and Larocco, 2008; Vogel et al., 2013; van Dam et al., 2015). Future work in the area will benefit from adopting a symptom-specific dimensional approach to assessment, distinguishing experiential from expressive negative symptoms (Kirkpatrick et al., 2011).

The findings of studies examining the specificity of the impact of trauma on psychosis will depend on the analytic approach employed, whether the relationships within victimization types and psychosis outcomes are considered, and if other difficulties, such as depression, are viewed as co-occurring mental health outcomes or mechanisms by which psychosis may arise. The debate is further complicated by the phenomenological overlap in traumagenic mental health problems, and that psychotic experiences, such as voices and paranoia, are relatively common in other diagnoses, including depression, bipolar affective disorder, PTSD and personality disorder (Johnson et al., 1991; Brewin and Patel, 2010; Schroeder et al., 2013; Cloitre et al., 2014; McCarthy-Jones and Longden, 2015; Geddes et al., 2016; Palmier-Claus et al., 2016; Kelleher and DeVylder, 2017; Okkels et al., 2017). Adopting a transdiagnostic, network analytic approach, examining potential causal systems of interacting symptoms, to understand the interplay between victimization, posttraumatic stress reactions and psychosis seems a promising way forward (Looijesetijn et al., 2015; Isvoranu et al., 2016, 2017).

## Current Understanding of the Relationship Between Trauma and Psychosis

Seminal cognitive behavioral models of psychosis addressed how trauma might shape beliefs, intrusions, appraisals and coping responses (Garety et al., 2001; Morrison, 2001). Central to these models is the proposal that the triggering of a biopsychosocial vulnerability gives rise to sensory-perceptual intrusions, which leads to a search for meaning (Maher, 1974). It is these attributions of meaning that result in psychotic experiences, which are then maintained by attempts to cope. For example, if a person’s childhood is characterized by physical abuse and strict religious observance, they may develop beliefs that spiritual entities are very powerful and that others will harm them. If they then experience a mugging, leading to distress and disrupted sleep, this could trigger an intrusion of a threatening voice saying ‘you’re going to get it’. In this context, it is understandable they might interpret the voice as a sign of persecution by evil spirits. This could then lead to hypervigilance, rumination and interpersonal avoidance, which may paradoxically perpetuate hearing voices through attentional focus, confirmatory bias and reduced opportunities to feel safe around others. Whilst the Garety et al. (2001) and Morrison (2001) models have been highly influential in research and practice, they arguably do not consider in detail the processes that may give rise to intrusions occurring in the context of trauma.

Morrison et al. (2003) further expanded these accounts, outlining three routes between trauma and psychosis: trauma may lead to psychosis, psychosis and related experiences can themselves give rise to PTSD, and that psychosis and PTSD may lie on a spectrum of shared reactions to trauma. They propose an integrative model of the spectrum of trauma reactions, emphasizing how the interpretation of intrusions determines the subsequent labeling of difficulties as PTSD or psychosis, and their consequences. In turn, appraisals of intrusions may be influenced by memory processes, dissociation and, schematic and metacognitive beliefs about intrusive experiences and coping responses. To return to the above example, if the person recognized the voice content as being related to what the perpetrator said during the mugging, the intrusion might instead be appraised as trauma-related. In this case, it would be viewed as a memory-based re-experiencing characteristic of PTSD instead of an externally attributed voice reflective of psychosis. A strength of this integrative model is in highlighting the potential spectrum from posttraumatic stress to psychosis. However, it does not consider in detail the diverse phenomenology of intrusions in psychosis and adopts a somewhat categorical approach to the appraisal of intrusions (i.e., that they will be attributed to either PTSD or psychosis). Whilst memory is mentioned as a potential process that might impact intrusions and how they are explained, the specific ways in which memory might shape intrusions and their interpretations is not explored.

Another important account, by Mueser et al. (2002), views PTSD and psychosis as distinct entities, and adopts a pragmatic position emphasizing how their symptoms may directly and indirectly interact to exacerbate each other. The model is descriptively useful at a symptom level, although unlike the previously mentioned models does not comment on the causal mechanisms that may give rise to PTSD and psychosis. Since these initial proposals, a number of theorists (Steel et al., 2005; Fowler et al., 2006; Longden et al., 2011; Read et al., 2014; Barker et al., 2015; Berry and Bucci, 2015) and empirical studies have added additional insights. A range of specific processes have been highlighted in recent literature, including stress sensitivity, dissociation, attachment styles, social defeat, trauma-related beliefs, contextual integration, and intrusive memories (e.g., Gracie et al., 2007; Varese et al., 2012a; Sitko et al., 2014; Alsawy et al., 2015; Geddes et al., 2016; Jaya et al., 2016; Valmaggia et al., 2016; Bucci et al., 2017). Broadly speaking, the processes implicated relate to (1) attempts to regulate emotion and survive trauma, (2) the storage and retrieval of trauma memories and (3) the meaning derived from these memories in the form of schema, beliefs or appraisals. However, this work has not yet been synthesized into a multifactorial model.

In real world settings, the lack of integration of these findings into a coherent framework is problematic, because people with psychosis and clinicians are faced with trying to understand and manage a complex and diverse array of posttraumatic stress reactions. Rates of diagnostic PTSD in psychosis are relatively modest and do not account for the full range of posttraumatic reactions that people experience. There is also variability in the extent to which psychotic experiences appear objectively or subjectively related to traumatic events, and this variation requires explanation. For example, people sometimes report their psychosis is unrelated to their trauma history, even though to others there may appear to be links between them. Further, people’s difficulties may lend themselves to distinct intervention approaches if they arise from different underlying mechanisms. A model integrating the current literature is clearly needed to address these practical and conceptual challenges, which can then guide formulation and intervention.

## A Model of Posttraumatic Stress in Psychosis

**FIGURE 1:**
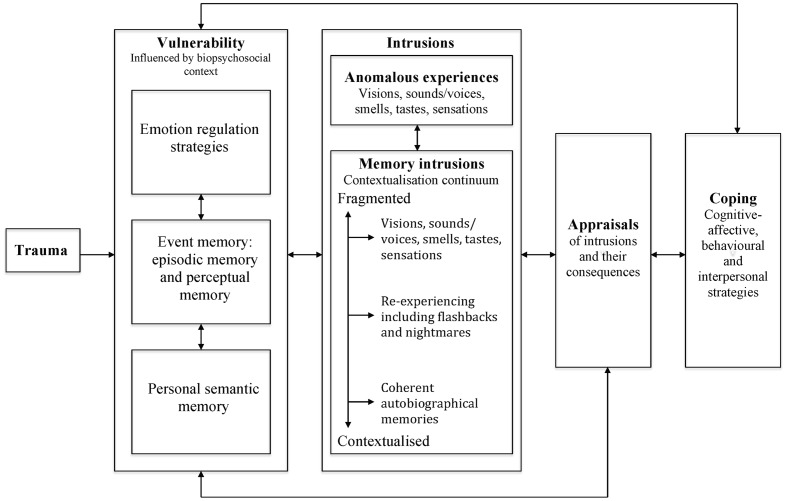
**A model of posttraumatic stress in psychosis**.

Drawing on the accounts outlined above and more recent findings in the area, a model of posttraumatic stress in psychosis will be described, based on cognitive-behavioral, attachment and neuropsychological perspectives of psychosis and PTSD. Specifically, trauma-related emotion regulation strategies and autobiographical memory are proposed to shape the phenomenology of intrusive imagery and subsequent appraisals and coping responses (see **Figure 1**). Distressing emotions are not explicitly named in the model as they are viewed as interwoven in all of the implicated processes, given the well-established role of emotional experience as a precursor to, part of and consequence of psychosis and PTSD (Birchwood, 2003; Freeman and Garety, 2003; Kessler et al., 2011; Fowler et al., 2012; Okkels et al., 2017).

### Vulnerability Factors

It is proposed in the model that one or more of three trauma-related vulnerability factors (i.e., emotion regulation, event memory (including perceptual and episodic memory representations) and personal semantic memory) give rise to a range of sensory-perceptual involuntary intrusions, and shape the appraisals of these and the subsequent coping responses. The theoretical basis for the vulnerability factors will first be described, with evidence for their impact on psychosis detailed in the “Intrusions,” “Appraisals,” and “Coping” Sections.

#### Emotion Regulation

The first psychological vulnerability factor proposed to lead to the development and maintenance of trauma-related psychosis consists of the ways in which people regulate emotions associated with threat resulting from traumatic events. Read et al. (2014) propose exposure to childhood victimization results in neurodevelopmental changes such as hyperactivity of the hypothalamic-pituitary-adrenal (HPA) axis. This leaves people vulnerable to understandable but unhelpful ways of regulating stress, which may then contribute to psychosis. We respond to threat with varying degrees of sympathetic (i.e., fight and flight) and parasympathetic (i.e., flag and faint) nervous system activation (the defense cascade), depending on the nature of the event and the individual (Schauer and Elbert, 2010). In the context of repeated childhood victimization, people may repeatedly oscillate through cycles of these reactions such that their response to the world is increasingly mediated by defense cascade responses. Dissociative detachment or ‘shutting down’ may be the most likely way of surviving inescapable threat, leading to the parasympathetic system being more persistently activated (Brown, 2006). These protective responses may therefore become habitual means of regulating emotion. In this sense, they can be viewed as attempts to survive extreme circumstances where fundamental developmental needs are not met (e.g., for safety, connection and autonomy) (Young et al., 2003).

The defense cascade is primarily conceptualized as reflecting cognitive-affective and behavioral responses. However, it is proposed that when these emotion regulation strategies become implicit ways of regulating threat, they will also show themselves in how the person relates to others (Bowlby, 1969, 1973). Parallels can therefore be drawn between emotion regulation and attachment styles (Hesse, 2008). For example, sympathetic nervous system activation could result in anger or fear, with congruent cognitive processing (e.g., hypervigilance and threat-focused reasoning), and aggressive, dependent or actively avoidant behavior and relating styles (Bartholomew and Horowitz, 1991). In contrast, dissociative detachment response (ranging from emotional numbing to more intense experiences of derealization and depersonalization) and interpersonal passivity may be manifestations of parasympathetic nervous system activity (Brown, 2006; Vogel et al., 2013). Thus, childhood victimization may result in increased or reduced stress sensitivity, or fluctuations between the two (Main and Hesse, 1990). As will be described in detail in the Sections on “Intrusions” and “Coping,” these strategies can paradoxically increase an individual’s sense of threat by impacting on intrusions and coping responses, thereby perpetuating psychosis.

#### Event Memory

The second vulnerability factor is memory for victimization events, which forms part of the autobiographical memory system^2^. In this model, autobiographical memory is defined as memory relating to life experiences that contributes to a person’s sense of self and includes three levels of representation. *Personal semantic memory* consists of abstracted meaning about the self, others and the world, which is derived from specific episodic memories and general sociocultural knowledge. *Episodic memory* consists of declarative, contextualized and allocentric event representations that can be manipulated, and voluntarily and involuntarily retrieved. *Perceptual memory* consists of detailed, egocentric, viewpoint dependent representations of sensory-perceptual stimuli experienced during events. Perceptual memory is held to be inaccessible to intentional retrieval independent of episodic or personal semantic memory representations but may, under certain conditions, be involuntarily recalled. Perceptual and episodic representations are relevant to understanding the storage and retrieval of event memory, and will be considered below, with the role of personal semantic memory as a vulnerability factor outlined in the next section.

So, how do we store and recall memory for events, and how do emotions experienced during trauma impact on event memory? Memory researchers propose that long-term memory for specific, everyday events is primarily a function of episodic memory, processed in the structures of the medial temporal lobe, including the hippocampus, and prefrontal areas (Tulving et al., 1991; Conway and Pleydell-Pearce, 2000; Brewin, 2001, 2014; Conway, 2001, 2005, 2009; Brewin et al., 2010). When an event is experienced, sensory-perceptual stimuli (including emotions, sights, sounds, sensations, tastes and smells) are initially encoded in perceptual memory through subcortical structures such as the amygdala. This encoding is then converted into a more abstract representation in episodic memory, consisting of a contextual representation of spatio-temporal information (of when and where stimuli were experienced), which in turn is integrated into personal semantic memory.

In everyday situations, the sensory-perceptual information in perceptual memory fades once it has been represented in episodic memory, and becomes relatively inaccessible. However, the event memory is still available for voluntary retrieval. When a person tries to recollect what happened, a search leads to the activation of the abstract representation in episodic memory, which then reconstructs the associated perceptual representation. This memory will be experienced as sufficiently vivid to conjure a sense of recollection, but less intensely than when the event actually happened, so it is perceived as a past experience. For example, if someone is asked what happened on a recent shopping trip, this will trigger an iterative search of his or her memory until the specific episodic representation of the event is identified. The abstract, episodic representation will then be used to reconstruct the perceptual experiences associated with the shopping trip so the person can recollect what happened.

Critical to understanding the role of victimization in psychosis is the proposal that, in the context of increased emotion, representations in perceptual memory may be encoded in greater detail than usual (Ehlers and Clark, 2000; Hackmann and Holmes, 2004; Brewin et al., 2010). The consequence of this is that when the episodic memory is later retrieved, the sensory-perceptual stimuli present at the time of the event (stored in perceptual memory) will be experienced more intensely than if encoded under less emotional conditions. For example, if a person accidentally urinated in public during a shopping trip, the anxiety and shame experienced as a result would be encoded in detail in their perceptual memory. When the memory is recalled it would still be experienced as a past recollection, but the detailed perceptual representation would result in the emotions felt at the time being acutely experienced in the present moment.

Further, cognitive-behavioral models of PTSD propose that, under conditions of extreme emotion, the episodic processing of information may also change. The encoding of contextualized representations is inhibited (also known as C-reps, arising from conceptual processing; Ehlers and Clark, 2000; Brewin et al., 2010) and, instead, stimuli are encoded in even greater detail in perceptual memory (also known as sensory representations, or S-reps, driven by data driven processing; Ehlers and Clark, 2000; Brewin et al., 2010). This has an evolutionary advantage as it supports faster processing of information and activation of survival responses, and also ensures detailed information about threat is available after danger has passed, when important learning can be integrated into memory (LeDoux, 1998).

However, event memories encoded under conditions of extreme emotion are retrieved differently from less emotional memories. First, given their reduced contextualization in episodic memory, perceptual memories are highly vulnerable to being directly retrieved by internal or external stimuli reminiscent of the highly arousing event. When this happens, they will be experienced (to a greater or lesser extent) as vivid and occurring in the ‘here and now’. This type of memory retrieval therefore reflects the hallmark re-experiencing symptoms of PTSD. Second, intentional recall of events is impaired, as the associated episodic memory has not been fully elaborated, particularly for moments of highest arousal during events (known as hotspots, Grey and Holmes, 2008). Thus, if a person experienced intense arousal due to witnessing a violent, armed robbery during a shopping trip, the episodic encoding of this event would be inhibited, whilst the storage of sensory-perceptual experiences would be enhanced. This would mean the person would have difficulty intentionally recalling a coherent account of the robbery, particularly peripheral details. They would also be vulnerable to experiencing intrusions, for example, of seeing people bleeding and feeling very scared, triggered by reminders of the event (e.g., going shopping, seeing men, ruminating about how they could have been killed). Intrusions encoded under circumstances of extreme emotion can become less intrusive if people are able to experience their memories, so the perceptual representations fade and become integrated into episodic memory. However, if people avoid their memory intrusions, the contextualization of episodic memory is prevented and involuntary intrusions will be maintained.

Habitual emotion regulation strategies related to persistent victimization may be particularly problematic for the storage and retrieval of distressing events. Whilst these strategies may be protective during trauma, they may exacerbate disruptions in episodic memory processing. If persistent, they will then prevent the contextual elaboration of perceptual memories into episodic memory representations, leading to intrusion maintenance. Indeed, Steel et al. (2005) and Fowler et al. (2006) argue that people affected by psychosis may have a weakened ability to integrate contextual information, possibly due to enhanced stress sensitivity. This is likely to further disrupt memory processing and the contextualization of perceptual representations in episodic memory (Read et al., 2014). People with a vulnerability to psychosis may therefore have perceptual memories that are even more fragmented from episodic memory. Plausibly, contextual representations could be absent at storage or retrieval such that the involuntary retrieval of perceptual memory is experienced with no awareness of its association to a past traumatic event. In this circumstance, the witness to the armed robbery who experiences intrusions of people bleeding and feeling scared may view these, not as memories, but as a premonition of a future attack. The potential role of extremely decontextualized memory intrusions in psychosis will be explored further in the Section “Intrusions: Memory Intrusions.”

#### Personal Semantic Memory

The third vulnerability factor for trauma-related psychosis is personal semantic memory, which reflects autobiographical knowledge that is abstracted from a specific time or place, and is likely mediated by the neocortex (Prebble et al., 2013). Personal semantic memory can therefore be viewed as reflecting the beliefs, schemas or appraisals implicated in cognitive-behavioral models of psychosis and PTSD, which can maintain problematic event memory representations (Ehlers and Clark, 2000; Garety et al., 2001; Morrison, 2001; Brewin et al., 2010). Alternatively, the cognitive-affective representations of the self and others in personal semantic memory also have parallels with the internal working models outlined by attachment theory (Bowlby, 1969, 1973). Personal semantic memory is derived from both episodic memories and broader sociocultural knowledge, and in this sense may be a proximal cognitive-affective mechanism by which risk factors associated with social defeat impact on psychosis (Selten et al., 2016). Personal semantic memory can be retrieved in concordance with or in isolation from episodic and perceptual memories; meaning it may be activated as abstract knowledge divorced from event content or in parallel with event memories to produce a rich, autobiographical experience.

Conway and Pleydell-Pearce (2000), Conway (2001, 2005, 2009), and Conway et al. (2004) propose personal semantic memory is categorically organized into general events, life periods and life stories, and may be particularly derived from key self-referent or self-defining episodic memories (Singer and Salovey, 1993). It is part of a self-memory system (SMS) dependent on frontotemporal regions of the brain. The SMS consists of personal semantic memory and a working self. The working self is a goal-orientated conductor of memory storage and retrieval that organizes both personal semantic and episodic memory in line with goals, to support problem solving and planning. The activity of the working self dynamically leads to and is shaped by personal semantic memory to derive an abstracted representation of the self and world. In this regard, personal semantic memory is held to be particularly central to a coherent sense of self (James, 1890; Prebble et al., 2013). A key function of the working self is to maintain correspondence and coherence between internal and external reality, and current goals. It therefore modifies memory encoding and retrieval to meet these needs. As stability of the SMS is prioritized this potentially gives rise to distortions in memory, as representations are modified to be congruent with the goals of the working self. The SMS thus has a key role in the reconstructive nature of memory. It may account for modifications in memory over time, as it transforms to fit with the person’s current goals or concerns (Conway and Loveday, 2015).

Childhood victimization will understandably shape personal semantic memory and the working self. Given these systems are resistant to change, they therefore provide a means through which victimization can have a pervasive impact on how people experience and interpret the world. A threat-focused SMS will give rise to congruent storage and recall of event memories. For example, a person who has the appraisal ‘I am bad’ represented in their personal semantic memory, based on memories of what their abusers said about them, would be more likely to retrieve episodic and perceptual representations in line with this appraisal, and to store aspects of new experiences which are consistent with this view of themselves.

### Intrusions

It is proposed that the trauma-related vulnerability factors outlined above are likely to give rise to certain types of intrusive imagery that, dependent on how they are appraised and responded to, may then lead to psychosis. The importance of imagery in understanding mental wellbeing, including psychosis, has long been recognized (Beck, 1970; Lang, 1977; Garety et al., 2001; Morrison, 2001; Hackmann and Holmes, 2004). Images are defined as “contents of consciousness that possess sensory qualities as opposed to those that are purely verbal or abstract” (Hackmann et al., 1998, p. 301) and can occur in any sensory modality. They are experienced “on a continuum from the near veridical reconstruction in the mind of a real event to the construction of an entirely hypothetical situation” (Martin and Williams, 1990, p. 268), and in this sense may be past or future orientated. Intrusive imagery is common, and so is viewed as a normative process, with distress and functional impact determined by responses to it (Hirsch and Holmes, 2007; Brewin et al., 2010). Transdiagnostically, it appears people usually experience a relatively small number of recurrent images, they tend to be thematically linked to people’s concerns, and associations with specific adverse events may not be recognized until people are encouraged to consider any links (Hackmann and Holmes, 2004). In the context of psychosis, the most studied type of imagery is voice hearing, although hallucinations in other modalities and multimodal experiences also occur and are increasingly subject to investigation (McCarthy-Jones et al., 2012; Woods et al., 2015). Other studies have noted that images are often associated with psychotic experiences, although there has been relatively little investigation of their phenomenology and relationship to memory (Morrison et al., 2002; Schulze et al., 2013; Ison et al., 2014; Sheaves et al., 2015). In this model of posttraumatic stress in psychosis, two types of trauma-related intrusive imagery will be considered. Anomalous experience intrusions influenced by emotion regulation strategies and autobiographical memory, and trauma memory intrusions arising from varying degrees of retrieval of personal semantic, episodic and perceptual memory representations. It is suggested that intrusions are likely to trigger each other, and so may act synergistically in the development and maintenance of psychosis.

#### Intrusions: Anomalous Experiences

The first type of intrusions, anomalous experiences, are proposed to arise through a number of putative pathways, involving emotion regulation (i.e., hyperarousal and dissociation) and representations in autobiographical memory. The rationale for and evidence for each of these will be considered.

In relation to hyperarousal, a hypervigilance subtype of voice hearing has been previously proposed (McCarthy-Jones et al., 2012, 2014; Garwood et al., 2015) and within this model is viewed as linked to the sensitization of the sympathetic nervous system following childhood victimization. Understandable hypervigilance to danger may result in a reduced threshold for threat detection in environmental noise (e.g., perceiving background sounds of people talking or traffic as a sign of danger), leading to intrusions of anomalous experiences. Previous evidence has focused on threatening content arising from the external environment, although it seems feasible this process could also relate to internal stimuli, such as detecting intrusions of somatic pain or felt-sense presences (McCarthy-Jones et al., 2014). In line with a role for trauma-related hypervigilance in anomalous experiences, Hardy et al. (2016) found posttraumatic hyperarousal mediated the association between childhood sexual abuse and voices in 228 people with relapsing psychosis. However, this study was cross-sectional and therefore the temporal association between hypervigilance and voices was not established.

Dissociative detachment is also proposed as a driver of anomalous experience intrusions. Habitual dissociation in an attempt to manage threat may paradoxically give rise to intrusive experiences. This is because it has a detrimental impact on the integration of sensory-perceptual processes, and so may result in intrusions into consciousness (Brown, 2006). The role of dissociation in intrusive anomalous experiences is supported by observed correlations between depersonalization/derealization and hallucinatory experiences (Vogel et al., 2011; Alderson-Day et al., 2014; Pilton et al., 2015), its mediation of the relationship between childhood trauma and hallucinations (Perona-Garcelán et al., 2012; Varese et al., 2012a; Hardy et al., 2016) and its prediction of hallucinations in the flow of daily life (Udachina et al., 2014).

The final way in which anomalous experience intrusions are proposed to arise is through the impact of autobiographical memory and the working self on current and future-orientated imagery (Brewin et al., 2010). It is proposed that autobiographical memory representations of childhood victimization may indirectly shape anomalous experiences, as the working self draws on personal semantic, episodic or perceptual memory representations to generate novel imagery. This will be affectively and/or perceptually congruent with the underlying representations. For example, a person who has been the victim of past harassment may experience an intrusive image of being humiliated at an upcoming social event. The perceptual qualities of this image (sensation of cheeks flushing, tension and physiological arousal) together with the associated appraisal (others will judge me) will be shaped by their personal semantic and event memories, even though the person may not have an explicit awareness of the link between this image and their past experiences.

Novel images generated from the working self and autobiographical memory representations will be shaped by victimization experiences and in this regard may map onto the inner speech subtype of voices proposed by McCarthy-Jones et al. (2012) and McCarthy-Jones et al. (2014). Evidence in support of this mechanism comes from studies finding that the autobiographical context of intrusions can be present without subjective awareness, and the observation that hallucinatory content is often thematically linked with victimization and personal goals, suggesting a possible influence of autobiographical memory representations and the working self (Hardy et al., 2005; Corstens and Longden, 2013; McCarthy-Jones et al., 2014; Varese et al., 2016).

Finally, it is also important to note that all anomalous experience intrusions may, if sufficiently arousing, be encoded as a long lasting memory representation and potentially intrude through the mechanisms outlined below. For example, a person who was neglected and bullied may be hypervigilant for threat and have an appraisal “I am dirty” in their personal semantic memory. This could lead to the generation of novel olfactory and auditory imagery in the form of the smell of feces and a voice saying, “you smell so bad, no one wants to know you.” The distress associated with this experience could lead to disruptions in autobiographical memory processing, with the perceptual representation stored in detail and the contextual, episodic representation inhibited, so the memory of the voice and the smell could later be involuntarily re-experienced.

#### Intrusions: Memory Intrusions

The second type of intrusions proposed to result from the trauma-related vulnerability factors are those arising from the direct retrieval of autobiographical memories. These are hypothesized to fall along a continuum of contextual integration. The position of intrusions is relatively rather than absolutely defined. For the purposes of the model, three distinct positions along the continuum are highlighted, although there will be variation within these positions as to the degree of contextualization experienced for any given intrusion. The positions range from fully contextualized memory intrusions, to those lacking in contextual integration (as is observed in the re-experiencing symptoms of PTSD), to at the extreme end, severely decontextualized memories that may be particularly characteristic of psychosis.

It is emphasized that, at this time, the continuum is speculative. It is not clear whether such a continuum exists or, rather, whether memory intrusions may be more accurately conceptualized categorically, experienced either as occurring in the ‘here and now’ or as past memories (Brewin et al., 2010). However, this dimensional approach to intrusions may better reflect the dynamic and reconstructive nature of memory, including how intrusions can be experienced with varying degrees of awareness of their link to past events. The mechanisms by which each of the three hypothesized types of memory intrusion arises will be considered below.

##### Contextualized memory intrusions

Intrusions of emotionally salient, contextualized memories may occur through two routes (Brewin et al., 2010). First, perceptual memories formed by emotionally arousing experiences may be retrieved by emotional or sensory cues, and given a context by corresponding information in episodic memory. Alternatively, a top–down process may occur when episodic memories are triggered with their associated perceptual memory. For example, if a person is dwelling on thoughts of being worthless, they might involuntarily remember a time when they failed. The contextual specifics of this event would be retrieved from episodic memory (being expelled from school), together with the corresponding perceptual memory of the stimuli present during the time (sinking sensation in stomach, welling up and sadness). Intrusions arising from this mechanism do not represent a shift in the nature of memory processing, but rather the impact of emotion on the storage and retrieval across the whole autobiographical memory system. There has been relatively little naturalistic investigation of this type of memory intrusion in psychosis, however, indirect support for the presence of this mechanism comes from findings indicating intrusive, coherent memories occur transdiagnostically and are likely to arise in the context of verbal, depressive rumination (Pearson et al., 2008; Brewin et al., 2010).

##### Re-experiencing memory intrusions

Re-experiencing memory intrusions, the ‘hallmark’ symptoms of PTSD, arise in the context of disruptions to the storage of event memory, as previously outlined. Detailed encoding in perceptual memory, coupled with inhibition of episodic representations, will increase the likelihood of representations of stimuli present at the time of trauma being triggered by associated cues (Brewin et al., 2010; Brewin, 2014). When people experience this type of intrusion, memory retrieval occurs, at least to some degree, in isolation from broader autobiographical recollection and the episodic grounding of events, such that they are experienced with a sense of occurring in the ‘here and now’ (Brown, 2006). However, the contextual, episodic representation is not entirely inaccessible, so this type of intrusion will still be experienced as linked to the associated event. Support for the role of re-experiencing in psychosis comes from findings indicating increased rates of PTSD in psychosis compared to the general population (de Bont et al., 2015), results from a non-clinical study that re-experiencing may partially account for the association between childhood sexual abuse and hallucinations (Gracie et al., 2007), and an association between posttraumatic intrusions and hallucinations in a large-scale population survey (Alsawy et al., 2015).

##### Fragmented memory intrusions

The final type of intrusions highlighted on the contextualisation continuum are fragmented sensory-perceptual experiences arising from perceptual memory representations. Together with the proposed anomalous experience intrusions, these memory intrusions are those that are most likely to reflect the hallucinatory imagery characteristic of psychosis. The theoretical basis for this type of intrusion, as highlighted earlier, is the proposal that people with psychosis may have a weakened ability to integrate contextual information, possibly linked to the sensitization of threat regulation (Steel et al., 2005; Fowler et al., 2006; Waters et al., 2006). Previous investigations of this hypothesis have highlighted that people high in schizotypy report more frequent, distressing intrusions after watching a traumatic film and following a road traffic accident (Holmes and Steel, 2004; Steel et al., 2008), and that there may be an association between contextual memory ability and intrusions in people high in psychosis proneness (Glazer et al., 2013). Steel and colleagues’ proposal therefore focuses on how contextual integration difficulties increase intrusion frequency, with other factors (e.g., paranormal beliefs) determining the appraisal of these intrusions and whether they lead to psychosis.

This new model takes the contextual integration hypothesis a step further to propose perceptual memory intrusions may occur in the absence of any episodic context, such that they are experienced as occurring in the ‘here and now’ with no recollection of their link to past trauma. In this case, it is the intrusion itself that will manifest as a hallucinatory, psychotic experience, regardless of how it is appraised. Nonetheless, this type of intrusion seems likely to compel the person to attribute it externally. For example, if a person experiences an intrusion of vaginal pain with no recollection of its association to the pain experienced during a rape, it makes sense they appraise this as an experience of being raped in the present moment by an evil spirit. It is acknowledged that investigating this hypothesis is methodologically fraught, as by definition these fragments of memory will not be viewed as linked to memory, which makes assessment of them difficult. Further, such intrusions may be phenomenologically impossible to distinguish from the intrusive anomalous experiences outlined earlier.

However, there are some preliminary indications that this type of memory intrusion may exist. First, this intrusion type has some parallels with a previously proposed dissociative autobiographical subtype of voice hearing, whereby people report experiencing voices (not memories) that replay previously experienced content (McCarthy-Jones et al., 2014). Further, studies demonstrating links between the content of trauma and voices are possibly in line with the involuntary retrieval of perceptual memories shaping psychosis (Hardy et al., 2005; Read et al., 2005; Corstens and Longden, 2013; McCarthy-Jones et al., 2014). A recently completed study also found that self-reported fragmentation of intrusive memories was positively associated with the severity of hallucinations, but not paranoia, in a small sample of people with psychosis (Marsh-Picksley et al., in preparation). Whilst very tentative, this study is the first to indicate that fragmentation of perceptual memory intrusions may impact on psychosis. Further, anecdotal evidence suggests that people may recognize their intrusions as memory-related only after having the opportunity to reflect on and contextualize episodic memories for the associated events. For example, a threatening voice saying ‘I’m going to have you’ being recognized, following trauma-focused exposure therapy, as linked to what was said during a sexual assault, when previously it had not been perceived as memory-based.

### Appraisals

In line with cognitive-behavioral models of psychosis and PTSD, the way in which people appraise their experience of intrusions will determine their consequences (Ehlers and Clark, 2000; Garety et al., 2001; Morrison, 2001). Within this model, it is proposed that appraisals will particularly be influenced by personal semantic memory and the goals of the working self. Appraisals of intrusions will therefore be consistent with and play a role in maintaining representations of the self and others in personal semantic memory. Appraisals can be primary (i.e., interpretations of the intrusion) and secondary (i.e., the implications of the interpretation). For example, a whispering voice may be interpreted as a sign of persecution by the devil (primary) that the person views himself or herself as being too weak to cope with (secondary). Both of these appraisals will be shaped by personal semantic memory, such as, the person believing they are evil and incompetent. Evidence supporting the role of personal semantic memory in psychosis comes from studies investigating trauma-related beliefs and schema, social defeat and attachment. Research has found that trauma is associated with negative beliefs about the self, world and others in psychosis, and these at least partially account for the relationship with paranoia in non-clinical, clinical and epidemiological samples (Kilcommons and Morrison, 2005; Gracie et al., 2007; Kilcommons et al., 2008; Geddes et al., 2016; Hardy et al., 2016; Jaya et al., 2016; Wickham and Bentall, 2016).

Other work has investigated the concept of social defeat, often assessed by measures of appraisals, which within this model are viewed as being derived from representations in personal semantic memory. For example, van Nierop et al. (2014b) in a large epidemiological study found social defeat mediated the relationship between childhood trauma, the extended psychosis phenotype and clinically significant psychosis. Similarly, Valmaggia et al. (2016) demonstrated the prospective impact of social defeat on paranoia in people at Ultra High Risk for psychosis compared to a control group. UHR participants reported higher levels of social defeat appraisals, and following exposure to a virtual social interaction environment reported more paranoia than controls, predicted by baseline levels of social defeat. Given social defeat is viewed as being shaped by previous events, this study suggests people’s interpretation of experiences within the virtual environment was influenced by appraisals stored in their personal semantic memory.

As noted previously, attachment styles can be viewed as reflecting representations of self and others in personal semantic memory. Insecure attachment, and particularly disorganized attachment, is associated with childhood victimization, and more severe psychotic experiences (Gumley et al., 2014; Korver-Nieberg et al., 2014; van Dam et al., 2014; Wickham et al., 2015; Bucci et al., 2017). Findings highlighting the role of attachment styles in psychosis are therefore arguably consistent with the view that appraisals in personal semantic memory shape interpretations of intrusions to give rise to psychotic experiences. A final piece of evidence comes from a trial that found trauma-focused interventions aiming to update threat-related information in traumatic event memory reduced paranoia as well as re-experiencing symptoms (van den Berg et al., 2015). This finding is therefore in line with the view that paranoid appraisals arise from autobiographical memory, and that modifying these representations will improve paranoia.

### Coping

Building on the proposal that habitual emotional regulation strategies can arise in response to victimization, this section will review how these strategies may, in turn, impact on coping with intrusions and appraisals. As noted previously, these strategies may relate to activation or deactivation of arousal, and manifest cognitive-affectively, behaviorally or interpersonally. In relation to the role of hyperarousal, is well established that stress sensitivity is associated with psychosis (DeVylder et al., 2016). Further, associations between childhood trauma and negative reactivity to daily stressors have been found, together with links to more severe positive symptoms (Glaser et al., 2006; Lataster et al., 2010; Lardinois et al., 2011). More recently, people with psychosis and a history of childhood trauma reported experiencing elevated stress sensitivity and paranoia compared to controls when in a virtual reality social environment (Veling et al., 2016). Other findings in support of the role of hyperarousal include studies demonstrating threat-focused processing in psychosis in the context of childhood victimization. For example, Bendall et al. (2013) found that people with a history of trauma demonstrated selective attention to threatening stimuli compared to controls. The previously mentioned findings by Hardy et al. (2016), that hyperarousal mediated the association between childhood sexual abuse and voices are also in line with this hypothesis. However, the cross-sectional design meant it was not possible to identify whether hyperarousal acted as a precursor to intrusions and/or as a coping response to intrusions and appraisals.

Indirect support for the role of trauma-related emotion regulation in psychosis also comes from findings highlighting the role of sleep disruption and rumination in psychosis. These have both been implicated as maintenance factors in posttraumatic stress difficulties, as they may perpetuate problematic autobiographical memory representations and exacerbate intrusions (Ehlers and Clark, 2000; Williams et al., 2007; Brewin et al., 2010; Freeman and Garety, 2014; Ricarte et al., 2017). Substance use is another potential coping strategy, and interactions have been found between childhood victimization, cannabis use and the odds of experiencing psychosis (Morgan et al., 2014). Fast thinking habits, or rapid reasoning, may also reflect a way of coping with threatening intrusions and appraisals, and could be associated with hyperaroused emotional regulation (Garety and Hardy, 2017). However, an association between fast thinking and emotion in psychosis has not been demonstrated, although this may be due to assessments lacking ecological validity (Freeman et al., 2008).

All the previously reviewed studies highlighting a role for attachment styles and dissociation in psychosis are also consistent with the hypothesized role of emotion regulation in coping with psychosis. For example, insecure anxious and avoidant styles may be linked to threat-focused coping, and disorganized attachment may be particularly associated with dissociation as a coping response (Braehler et al., 2013; van Dam et al., 2014; Bucci et al., 2017). Powers et al. (2016) reported that avoidance and numbing mediated the relationship between childhood trauma and psychosis in a large sample, after controlling for demographic variables and trauma exposure. Finally, it is noted that avoidant coping may also manifest as experiential negative symptoms (such as anhedonia, avolition and asociality) as people withdraw in an attempt to cope with their psychotic experiences (Raffard et al., 2010).

## Pathways from Victimization to Psychosis: Vignettes

To illustrate the model, three vignettes will briefly be described, based on clinical cases modified to ensure confidentiality. They aim to highlight different pathways between victimization and psychosis that anecdotally appear common in clinical practice. They are not intended to reflect discrete categories or subtypes. Whilst it is plausible that subtypes of trauma-related psychosis do exist, further empirical work is needed to investigate this hypothesis. Each person’s situation has been simplified, with a particular focus on the intrusions that appear to be playing a key role in driving their difficulties. The terminology in the formulations has been adapted from **Figure 1** to highlight how the model can be modified to a person’s understanding of their situation.

### Tanya (see **Figure 2**)

**FIGURE 2:**
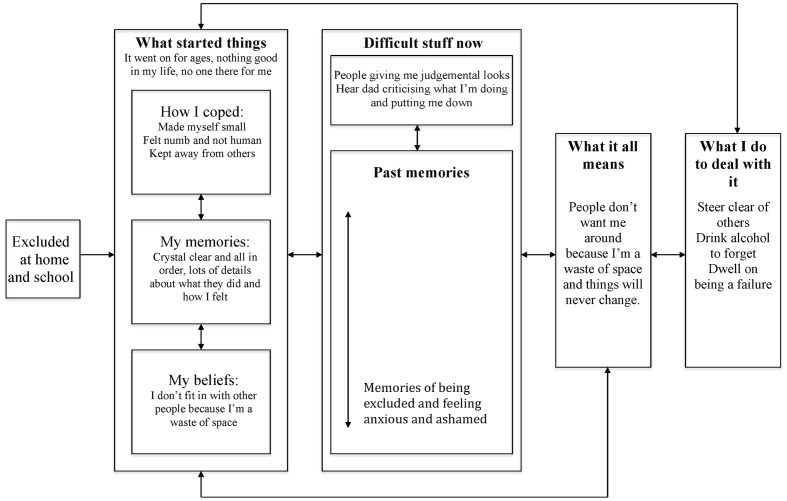
**Posttraumatic stress in psychosis model: Tanya’s formulation**.

Tanya described feeling very low and ashamed, as she believed others did not want her around because she was a waste of space. She reported things had always been this way, as growing up she did not fit in at home or school. Tanya said she intentionally made herself small so that others did not notice her, and became increasingly detached to the extent she felt numb and not human. It did not appear that she had experienced any periods of extreme arousal (e.g., characterized by overwhelming fear, horror or helpless) during her traumatic events of being excluded, although they had understandably triggered strong emotions of anxiety and shame. She reported being able to clearly recollect the most difficult events, with vivid impressions of what happened.

When around others, Tanya experienced them looking away from her and would be reminded of times when people had treated her similarly in the past. She also reported often hearing the voice of her dad criticizing what she was doing. Tanya described how these experiences confirmed her longstanding belief that people did not want her around because she was a waste of space, and that she did not think that things would ever change. She reported withdrawing and drinking alcohol excessively. She said she often ruminated on how much she had failed, and described a persistent, critical internal dialog.

In terms of formulating Tanya’s difficulties, it appears her early experiences gave rise to a pervasive avoidant style of relating to herself and others. This may have provided some protection from people and the intensity of her internal distress. She was able to coherently describe her past memories, with no avoidance, and did not report experiencing extreme arousal at the time of these events. This suggests her traumatic experiences had not been of sufficient intensity to disrupt contextual memory processing. Instead, she reported contextualized episodic memories, with vivid perceptual representations of what she had experienced at the time. The meaning of these experiences, that she was a waste of space had been ingrained in her personal semantic memory, and she was not able to recollect any alternative self-images.

In the context of this vulnerability, she was hypothesized to have both anomalous experience and trauma memory intrusions. The anomalous experiences took the form of perceiving others to be giving her judgmental looks and hearing a critical voice of her dad. It can be speculated that these experiences may have arisen from dissociative detachment and novel images generated from her autobiographical memory, in interaction with ambiguous stimuli in the environment (e.g., people looking at her). Memories of being excluded could have generated anomalous internal experiences of perceiving others looking at her judgmentally and a critical, derogatory voice. The salience of these experiences may have been further magnified by her persistent dissociation. The anomalous experience intrusions may have triggered and/or been exacerbated by intrusions of contextualized memories, such that Tanya would often have vivid recollections of the most difficult times from her past.

Understandably, these intrusions appeared to confirm her appraisal, represented in personal semantic memory, of herself as a waste of space. Further, her secondary appraisal, that things would never change, was in line with her lack of self-efficacy. Her coping responses were shaped by how she had learnt to survive growing up, with avoidance and rumination dominating, which in turn maintained her difficulties.

The formulation indicates that recommended cognitive-behavioral strategies may be of benefit, including developmental and maintenance formulations, psychoeducation, normalization, modifying coping, cognitive reappraisal, behavioral experiments and imagery modification (Morrison, 2017). In addition, the model specifically highlights the importance of addressing the impact of past ways of coping and memory recall on current problems. Following this, it appears imagery modification purely aiming to elaborate contextual representations in Tanya’s episodic memory, as in traditional exposure approaches, may not be helpful because her memory is already contextualized (Foa et al., 2007). Instead, it may be more useful to focus on developing new episodic representations of her intrusive memories, using techniques such as competitive memory training and imagery rescripting (Steel et al., 2015; Morina et al., 2017).

Further, whilst simple imagery transformations could be used to modify Tanya’s memory (e.g., imagining a time when she felt excluded disappearing or a negative outcome happening to those who left her out) it is possible that imagery modification may be more potent if the transformation addresses representations in both her event and personal semantic memory. This could involve, for example, having a trusted older adult enter the memory to remind her that she is lovable and worthwhile, even if others have not cared for her as they should. Given Tanya’s prolonged attachment difficulties, she may need support to notice or develop alternative experiences that can be drawn on when updating memories and images. Limited re-parenting and empathic confrontation, together with support to live a valued life through social inclusion, may assist with developing these new perspectives (Young et al., 2003).

### Kobe (see **Figure 3**)

**FIGURE 3:**
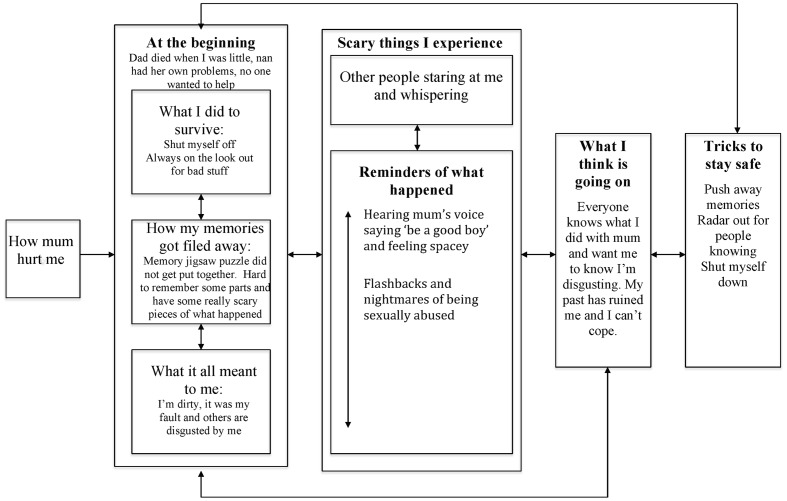
**Posttraumatic stress in psychosis model: Kobe’s formulation**.

Kobe reported his main difficulty was people staring and whispering about him. He explained how it was just him and his mother growing up, who hurt him by making them have sexual contact. Kobe said he would shut off during this abuse and pretend it was not happening. He lived in fear of it happening again and was always on the look out for ways to avoid it or escape. Kobe described how he was quite confused about some of what happened, and his memories from that period of his life were different to later times. He indicated how he always knew what happened was his fault as he was dirty.

Kobe said he could tell from others’ stares and whispers that they were disgusted by him and knew what happened. He also reported experiencing flashbacks and nightmares of being sexually abused. Kobe further described having a very scary experience of hearing his mum’s voice saying ‘be a good boy’ and feeling very spacey. He interpreted these experiences as confirmation that everyone knew about and was judging him for this past, and that his life had been ruined and he could not cope. He described how he protected himself by trying not to think about the past, having radar out for signs that people knew him, and shutting down.

A preliminary understanding of Kobe’s difficulties suggests he tried to cope with the abuse by alternating between being hypervigilant for signs that an assault would happen, and being dissociatively detached when it did and feeling shut off. Kobe’s confused intentional recall of the abuse suggests that his perceptual memory encoding could have been enhanced, with his episodic processing of events inhibited, due to the understandable extreme arousal he would have experienced. This would have meant that his memories for the assaults were poorly contextualized and vulnerable to being triggered by associated stimuli. It may be that Kobe’s shutting off during assaults, and being hypervigilant at other times, further exacerbated the lack of episodic processing. His perceptual memories might have then been even more fragmented from their episodic context. Kobe’s main appraisals of his experiences, represented in his personal semantic memory, were that he was dirty, it was his fault, and others would judge him negatively for what happened.

Against this background of vulnerability factors, Kobe had anomalous experience intrusions of people staring and whispering about him, possibly driven by hypervigilance. He was constantly alert for signs that people would know what had happened, so would likely have a lower threshold for detecting stimuli consistent with his fears. Kobe also experienced trauma memory intrusions in the form of flashbacks and nightmares of abuse, which made sense given the lack of contextualization of his trauma memory. The intrusive voice and spacey sensation Kobe experienced might have been manifestations of perceptual memories that were very fragmented from their episodic context, given they had content which appeared directly related to the abuse but were not experienced as arising from memory. Kobe experienced these intrusions not as posttraumatic stress difficulties but as further confirmation of others’ judging him for his past, in line with his appraisals in personal semantic memory. His tricks to stay safe would likely further perpetuate his difficulties. He constantly tried to suppress his intrusions, was alert and experienced dissociative detachment. This made it difficult for him to elaborate and contextualize his traumatic memories, to have the opportunity to evaluate the validity of his internal experiences and appraisals through developing safer relationships with others, and to consider other possible explanations for what might be happening.

As with Tanya, recommended cognitive-behavioral approaches for psychosis may support Kobe in managing his difficulties. However, in contrast to Tanya, his formulation suggests memory exposure is indicated, with the aim of elaborating and updating episodic memories so the perceptual representations are less likely to intrude. Possible techniques include narrative exposure therapy, prolonged exposure, reliving with restructuring, and eye movement desensitization and reprocessing therapy (Shapiro, 2001; Ehlers et al., 2005; Foa et al., 2007; Schauer et al., 2011). However, to date, little is known about the comparable effectiveness of these approaches in psychosis, and what factors, if any, should moderate intervention choice. It is also not clear whether exposure itself is sufficient to update the memory, or whether further cognitive or experiential reappraisal may be required.

A further dilemma in working with Kobe is how and when to introduce memory-focused interventions. Best practice guidelines recommend that trauma-focused therapy be offered. However, targeting past memories may not be a priority for Kobe. His main concern is people judging him negatively, even though the formulation suggests elaborating his memory may have a beneficial effect on his fears about others. As with any therapy, an open and transparent discussion of intervention options (including their pros and cons) is indicated to support Kobe in identifying his preferred approach. This would also involve developing a rationale for memory work that fitted within Kobe’s belief system. For example, framing the aim of exposure as being to minimize the impact of the past memories on him, so he might be more able to focus on managing his current difficulties with others.

### Mo (See **Figure 4**)

**FIGURE 4:**
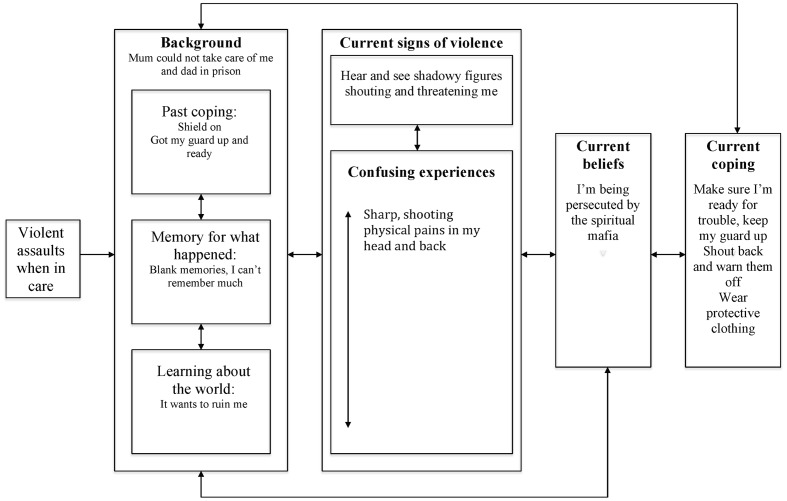
**Posttraumatic stress in psychosis model: Mo’s formulation**.

Mo’s main concern was about being persecuted by the spiritual mafia. He explained he was brought up in a children’s home where he frequently experienced violent assaults. Mo said he coped by having a shield on which meant he was not bothered much by what was happening, although he also learnt to have his guard up and was alert to signs of things kicking off. Mo was dismissive when asked about his experiences, and said he did not have much memory of what happened. However, he was clear that he had learnt early in life that the world wants to ruin him. He indicated that he now heard and saw shadowy figures shouting and being threatening, which would sometimes be accompanied by physical pains. Mo described how he knew the spiritual mafia was persecuting him, as he was cursed and it made sense others would continue to assault him. He described how he still coped by having his guard up, being alert to signs of trouble, and trying to fight back. He also reported wearing protective clothing to reduce the likelihood of the spirits being able to hurt him.

In terms of understanding Mo’s difficulties, his shield could be viewed as an extreme form of dissociative detachment, as he reported little or no emotional response to the assaults. He also appeared to have become hyperviglance to threat, which could reflect a sensitization to stress. Intriguingly, Mo reported very little recollection of what had happened to him in the past. It is difficult to establish the extent to which this reflected an avoidance of memory retrieval and/or the nature of the underlying memory representation for what happened. It is possible that a shutting down of attention during the assaults meant that his episodic encoding of them was significantly limited such that he did have significantly impaired voluntary retrieval for the events. However, given the nature of what happened, he would have likely had at least some detailed perceptual representations of the assaults. Mo’s appraisal of his early experiences, represented in personal semantic memory, was that he knew the world wanted to ruin him.

Mo reported no memory intrusions, although he did have multimodal experiences of shadowy threatening figures and experienced intrusive pain. Establishing the mechanisms underlying these experiences is difficult, and alternative hypotheses should be considered. However, the shadowy threatening figures appear more thematically as opposed to directly related to previous assaults (as the content of what they said reflected current not past concerns) and he described them as being adult figures, not children or young adults. It is therefore hypothesized that these intrusive images might have arisen from his emotion regulation strategies of having his shield up and being on guard, together with his working self and autobiographical memory representations generating novel images that were thematically congruent with his previous experiences. However, it is noted that these intrusions could also arise from involuntary recall of memories modified over successive retrieval attempts, in line with the goals of his working self to be alert for danger.

In contrast, it is speculated that the somatic pain Mo experienced could be a very fragmented intrusion arising from perceptual memories of what he experienced during past assaults. Given his report of his trauma memories, it is potentially feasible that this perceptual memory fragment was so decontextualized when intruding it had no grounding episodic context. As with Tanya and Kobe, Mo’s appraisal of his intrusions was shaped by his personal semantic memory (i.e., that others want to harm him) and the anomalous nature of his intrusions, such that he viewed them as being a spiritual attack by the mafia. Trying to cope with this meant he was in a persistently hyperaroused state, increasing the likelihood of him noticing any possible threats, maintaining his appraisal of others in personal semantic memory, and preventing any possibility to reflect on his past.

Therapeutically, it is difficult to know whether trauma-focused interventions would be helpful for Mo. He reported no distressing impact of trauma although at the same time his current concerns appeared to be a mirror to his past. As with Tanya and Kobe, conventional cognitive-behavioral techniques targeting his appraisals and coping may help him in developing a sense of safety in life. In addition, at least some psychoeducation about trauma and its consequences appears indicated. This could include tentative exploration of Mo’s thoughts about whether this information is relevant to his situation and, if he wishes to pursue further, shared formulation about the ways his past may contribute to his current difficulties. It of course should be respected that Mo and others may never be willing or able to address their experiences of victimization, and other approaches can be effectively used to support moving on in life. Nonetheless, it is suggested it is helpful to hold trauma-related hypotheses in mind and ensure people are aware of the option of trauma-focused therapy, should they wish to pursue this in the future.

## Clinical Implications

A key implication of this model is that trauma and posttraumatic stress processes should be assessed in psychosis. However, evidence suggests routine assessment is rare in mental health services (de Bont et al., 2015; Brooker et al., 2016). This is particularly concerning in the UK, where it is recommended in best practice guidelines (National Institute for Health and Care Excellence [NICE], 2014). Improving practice requires the development of a trauma-informed service culture, clinicians having a biopsychosocial model of psychosis, skills training, and supervision of assessment and follow-up (Walters et al., 2016).

It is also important to recognize that assessment of posttraumatic stress in psychosis is complex. People often have a multitude of trauma, posttraumatic stress and psychosis experiences, with varying etiological origins. It can also be hard to disentangle types of memory encoding and retrieval, and establish whether difficulties are indeed trauma-related. Memory representations, posttraumatic stress reactions and psychosis will also change over time, influenced by a range of factors (particularly the reconstructive nature of trauma memory) and the potential to identify any links between them will oscillate accordingly. In addition, it is obviously not possible to directly assess stored memory representations; they can only be inferred from people’s report of intrusions and their voluntary recall. People’s reports will also be moderated by their ability to reflect on their memory processes and links with prior difficulties, which is a complex metacognitive task (McCarthy-Jones et al., 2014). Progress in the area will be supported by the development of more sensitive tools for assessing trauma memories (Brewin, 2015; Ford, 2016).

In terms of formulation, clinicians are encouraged to use the model flexibly and adapt to each individual. It is emphasized that in practice it would not be recommended, unless there is a clear preference from the person, to collaboratively discuss all aspects of the formulation given its complexity. Instead, the model is intended to guide clinicians in highlighting potential directions for collaborative micro-formulation and intervention. People may not find it helpful to differentiate intrusion types and in which case intrusions should be formulated generally, as in existing cognitive-behavioral formulations of psychosis (Garety et al., 2001; Morrison, 2001). The therapist, however, can hold in mind hypotheses about the distinct mechanisms contributing to different intrusions, and implications for intervention.

The model suggests trauma-focused therapy for psychosis should target emotion regulation and autobiographical memory. This proposal is consistent with protocols for cognitive-behavior therapy for psychosis and PTSD, given their focus on modifying coping, and understanding and contextualizing beliefs through experiential learning (e.g., Fowler et al., 1995; Ehlers and Clark, 2008; Morrison, 2017). This model builds on these approaches to emphasize the developmental context of coping mechanisms and indicates memory interventions should be prioritized, at least for some people. Further, as highlighted by the vignettes above, it suggests that different interventions should be used depending on the nature of people’s intrusions and the hypothesized mechanisms driving them.

However, it may also be the case that the precise form of memory modification is less relevant. Instead, the key mechanism of change could be to develop alternative memory representations, through whatever means, that have the retrieval advantage over distressing representations (Brewin, 2006). For example, there are indications that imagery rescripting may be beneficial in PTSD, even though it does not involve fully recontextualising the perceptual memory representation, and instead focuses on encoding a new, less distressing representation (Morina et al., 2017). Interestingly, two recent trials of trauma-focused therapy for PTSD in psychosis found that exposure and EMDR, but not cognitive restructuring, were effective in reducing posttraumatic stress. This suggests the importance of accessing and experientially modifying event memories, not just focusing on appraisals in personal semantic memory (van den Berg et al., 2015; Steel et al., 2017b).

Another intervention consideration is whether trauma memories themselves need to be targeted or if it may be beneficial to engender ways of relating to internal experiences or memory retrieval that naturalistically support helpful processing. For example, initial findings suggest mindfulness or memory specificity training may reduce difficulties in people with experience of childhood trauma and current depression, and in PTSD, although to date this has not been robustly evaluated in psychosis (Hitchcock et al., 2016; Kuyken et al., 2016). An alternative approach would be to focus on emotion regulation. Learning new ways to manage threat may have a beneficial impact on memory representations, intrusions and appraisals. For example, third wave approaches including dialectic behavior therapy, mindfulness, acceptance and commitment therapy and compassion-focused therapy could be of value (Linehan, 1993; Mayhew and Gilbert, 2008; Morris et al., 2013). Further research is required to identify the effectiveness of these approaches, and what works best for whom.

Regardless of the specific cognitive-behavioral techniques used, as with any psychological therapy, a safe, secure and validating therapeutic relationship is essential, and the importance of humane values is emphasized (Brabban et al., 2016). Indeed, non-specific factors in the therapeutic relationship may be particularly valuable in providing an alternative experience to how people were treated in the past, and memories of this may usefully moderate threat-focused representations. Finally, whilst the model outlined here has focused on psychological mechanisms, the social context in which difficulties occur and the immense value of social and societal interventions is acknowledged. The social consequences of victimization, in terms of ongoing experiences of inequality and discrimination, are well documented and can of course significantly impede people’s potential to move on in their lives (Gilbert et al., 2010; Metzler et al., 2016). Addressing these consequences (including broader societal attitudes and responses to victimization) is essential and could, indirectly, have a beneficial impact on the posttraumatic stress processes outlined in the model.

## Future Research

To date, there has been relatively little investigation of the posttraumatic stress processes implicated in the model and clearly further work is needed to investigate the hypothesized causal pathways. A strength of the model is its mechanism-focused conceptualization, meaning it can cut across diagnostic boundaries. Whilst the focus in this article has been on psychosis, the model could potentially be used to formulate other posttraumatic stress presentations where intrusions are viewed as playing a causal role in maintaining distress. The model also has a developmental emphasis, with a focus on childhood trauma. However, it is recognized that trauma may occur later in life and/or be related to experiences of psychosis. The mechanisms described could be applicable to trauma occurring at any life stage.

The methodological challenges of researching the area are significant. It is ethically and legal complex and costly to conduct methodologically robust, prospective studies, and many studies use a cross-sectional design and relatively small samples. The complexity of pathways to psychosis, and the heterogeneity of psychosis itself, also clouds research endeavors. In addition to assessment issues raised previously, concerns have been highlighted regarding the reliability of trauma reports in psychosis. However, comparable reliability has been found to general population samples and obstacles to disclosure mean that false negatives are more of an issue (Hardt and Rutter, 2004; Fisher et al., 2011). A further concern is that subjective reports of intrusions may not accurately assess involuntary retrieval. Avoidance of trauma memory may distort people’s responses, and people may not be aware of intrusions when they are triggered. Whilst ethically contentious, it could be preferable to evoke intrusions under experimental conditions or to develop new techniques for *in vivo* investigation. Greater clarity and consensus in operationalizing and assessing dimensions of voluntary and involuntary memory retrieval, such as fragmentation and coherence, will also support progress in the field.

A possible starting point for developing our understanding of the pathways from trauma to psychosis could include detailed phenomenological assessment of involuntary and voluntary recall of traumatic memories, and other intrusions. For example, the model predicts more incoherent voluntary recall would be related to increasingly fragmented, vivid and frequent intrusions, which could manifest as hallucinatory experiences. Moving beyond diagnostic boundaries to focus on the causal interplay between specific posttraumatic processes and symptoms is also a useful direction for future research. Experience Sampling Methodology, whilst not without its limitations, may also be a more promising route to investigating the ways in which the implicated processes interact in real-world settings, and potentially provide a means to deliver intervention prompts (e.g., to support people in responding helpfully to intrusions) (Reininghaus et al., 2015).

## Conclusion

In conclusion, a multifactorial model of posttraumatic stress in psychosis has been outlined, drawing on current theoretical accounts and empirical evidence. Emotion regulation and autobiographical memory (including perceptual, episodic and personal semantic representations) are hypothesized to lead to the development and maintenance of intrusions, appraisals and coping responses in psychosis. Two types of intrusive experiences are proposed to account for the diversity of phenomenological links between trauma and intrusions. First, anomalous experience intrusions, driven by emotion regulation and the generation of novel images from autobiographical memory, may explain experiences which appear unrelated to or which only thematically mirror trauma history. Second, trauma memory intrusions may be retrieved at any point along a continuum of contextualization, with recollections ranging from coherent memories to very fragmented intrusions. Given the extent of their fragmentation, the latter are unlikely to be perceived as arising from memory events even if they seem objectively linked. The reconstructive nature of memory is emphasized, as stored representations may be modified over time depending on the goals of the working self. It is highlighted that at this stage the model is speculative, and robust empirical designs are needed to elucidate the mechanisms by which victimization impacts on psychotic experiences. However, it is hoped that the model may contribute to supporting people with psychosis and clinicians in understanding the sometimes overwhelming and complex difficulties that they face. In healthcare services, our role is to get better at recognizing when people have experienced trauma, assisting them in making sense of their problems and providing the support they need to move on in their lives.

## Author Contributions

AH had sole responsibility for the development and writing of the manuscript.

## Conflict of Interest Statement

AH is the Psychology Lead for Posttraumatic Stress in Psychosis, Psychosis Clinical Academic Group, South London and Maudsley NHS Foundation Trust. She provides consultation, supervision and teaching on posttraumatic stress in psychosis including payment from external agencies, and is involved in studies developing the theoretical understanding of and therapies for posttraumatic stress in psychosis.

## Footnotes

1 Trauma, traumatic events and traumatic experiences are used interchangeably. The considerable debate regarding the definition of traumatic stressors is acknowledged and beyond the scope of this article (Friedman et al., 2011). For the purposes of this paper, a broad definition of traumatic events is employed, defined as any event(s) the individual perceives as involving a significant physical and/or psychological threat to the self.

2 Different, albeit overlapping, conceptualizations of autobiographical memory have been proposed in the literature, with debate over the precise mechanisms involved (Conway and Pleydell-Pearce, 2000; Baddley, 2001; Brewin, 2001, 2014; Conway, 2001, 2005, 2009; Tulving, 2002, 2005; Brewin et al., 2010; Prebble et al., 2013). For the purposes of this model and ease of understanding, a pragmatic conceptualization is adopted. This is done with the acknowledgment that some theorists have used different terminology to describe the referenced concepts.
